# Expression of Oligodendrocyte and Oligoprogenitor Cell Proteins in Frontal Cortical White and Gray Matter: Impact of Adolescent Development and Ethanol Exposure

**DOI:** 10.3389/fphar.2021.651418

**Published:** 2021-05-06

**Authors:** Wen Liu, Aaron R. Rohlman, Ryan Vetreno, Fulton T. Crews

**Affiliations:** Bowles Center for Alcohol Studies, University of North Carolina at Chapel Hill, Chapel Hill, NC, United States

**Keywords:** NG2, glia, oligodendrocyte, progenitors, adolescent maturation, myelin, ethanol, alcohol

## Abstract

Adolescent development of prefrontal cortex (PFC) parallels maturation of executive functions as well as increasing white matter and myelination. Studies using MRI and other methods find that PFC white matter increases across adolescence into adulthood in both humans and rodents. Adolescent binge drinking is common and has been found to alter adult behaviors and PFC functions. This study examines development of oligoprogenitor (OPC) and oligodendrocytes (OLs) in Wistar rats from adolescence to adulthood within PFC white matter, corpus callosum forceps minor (fmi), PFC gray matter, and the neurogenic subventricular zone (SVZ) using immunohistochemistry for marker proteins. In addition, the effects of adolescent intermittent ethanol exposure [AIE; 5.0 g/kg/day, intragastric, 2 days on/2 days off on postnatal day (P)25–54], which is a weekend binge drinking model, were determined. OPC markers NG2+, PDGFRα+ and Olig2+IHC were differentially impacted by both age and PFC region. In both fmi and SVZ, NG2+IHC cells declined from adolescence to adulthood with AIE increasing adult NG2+IHC cells and their association with microglial marker Iba1. PFC gray matter decline in NG2+IHC in adulthood was not altered by AIE. Both adult maturation and AIE impacted OL expression of PLP+, MBP+, MAG+, MOG+, CNPase+, Olig1+, and Olig2+IHC in all three PFC regions, but in region- and marker-specific patterns. These findings are consistent with PFC region-specific changes in OPC and OL markers from adolescence to adulthood as well as following AIE that could contribute to lasting changes in PFC function.

## Introduction

Adolescent maturation of cognitive abilities and emotional responses parallels brain development of prefrontal cortical (PFC) networks across species (Spear, 2000; Dahl, 2004; Crews and Vetreno, 2016). Cortical myelination and white matter increase in adolescence, a trend that continues in young adulthood in both rodents (Ehlers et al., 2013a; Oguz et al., 2013) and humans (Gerig et al., 2011; Mills et al., 2016; Pfefferbaum et al., 2018). Although neurogenesis is largely complete by the beginning of adolescence, synaptic changes and myelination continue through adolescence and into mature adulthood with both likely contributing to development of adult executive function and emotional responses (de Graaf-Peters and Hadders-Algra, 2006; Semple et al., 2013; Spear, 2013). Interestingly, oligodendrocyte progenitor cells (OPCs) form early in development and continue to proliferate and form new oligodendrocytes (OLs) across adolescence and adulthood. Development of PFC includes increases in brain white matter volume reflecting myelination of axons. Myelination of PFC axons increases transmission velocity consistent with improved PFC function (McDougall et al., 2018), perhaps by inhibition of impulsive responses. Adolescent maturation in humans and rats includes reductions in PFC gray matter cortical thickness (Vetreno et al., 2017) and increases in corpus callosum white matter (Paus, 2005; Vetreno et al., 2016; Juraska and Willing, 2017). Human MRI studies find that volume of cortical gray matter peaks in early adolescence, then gradually declines in late adolescence to adult levels that, depending upon brain region, coincide with the development of intelligence or, in other individuals, with the onset of mental disease (Huttenlocher, 1984; Giedd et al., 1999a; Giedd et al., 1999b; Gogtay et al., 2004; Paus et al., 2008; Semple et al., 2013). Adolescent cortical gray matter thinning may involve synapse loss as well as myelin growth into cortical gray matter since corpus callosum white matter increases during maturation to adulthood (Reiss et al., 1996; Paus et al., 2008; Konrad et al., 2013). Similarly, white matter integrity investigated using diffusion tensor magnetic resonance imaging (DTI) is altered during adolescence in rats (Oguz et al., 2013; Vetreno et al., 2016) as well as humans, where it parallels maturation of cognitive ability (Lenroot and Giedd, 2006; Bava and Tapert, 2010; Fan et al., 2019). These findings prompted our investigation of PFC OPC and OL markers to better understand adolescent-to-adult maturation of OPCs and OLs.

OLs are formed in embryogenesis and early postnatal life from OPCs and persist long into adulthood, consistent with adolescent developmental maturation of white matter volume and structure involving maturation of myelin forming OLs during development to adulthood. We used immunohistochemistry (IHC) to investigate changes in OLs and OPCs in PFC gray and white matter. Two ages were studied, post-puberty late adolescence, i.e., rat postnatal day (P)57, which is equivalent to human 17–22 years of age (Semple et al., 2013), and mature adulthood, i.e., P95. Myelin-forming OLs express specific proteins responsible for myelin formation and structure that include myelin basic protein (MBP), proteolipid protein (PLP), myelin-associated glycoprotein (MAG), and myelin oligodendrocyte glycoprotein (MOG), all of which are thought to contribute to the structure of myelin formed by mature OLs (Gonzalez-Perez and Alvarez-Buylla, 2011; Marques et al., 2016). 2′,3′-Cyclic-nucleotide 3′-phosphodiesterase (CNPase) is a myelin-associated enzyme that makes up 4% of total brain myelin protein and is known to change with age (Hinman et al., 2008). We also assessed OL transcription factor proteins *Olig1* and *2* (Lu et al., 2002; Zhou and Anderson, 2002) that are expressed in myelin-forming OLs (Marques et al., 2016). We also investigated OPCs, which are continually expressed in adulthood, representing about 5% of brain cells (Dawson et al., 2003) and are identified by neuron-glial antigen 2 (NG2, the protein of the gene chondroitin sulfate proteoglycan 4 [CSPG4]) and platelet-derived growth factor receptor alpha (PDGFRα) (Schiffer et al., 2018). NG2+ cells are a resident population of brain glial progenitor cells that include OPCs but are distinct from neurons, astrocytes, mature OLs, and microglia (Nishiyama et al., 2009). OPCs form OLs in adolescence and adulthood, although the rate of proliferation and maturation declines in adulthood (Young et al., 2013). Further, gray and white matter mature in adolescence and have different rates of OL formation, so we investigated PFC white matter in corpus callosum forceps minor (fmi), as well as two PFC gray matter regions, prelimbic cortex (PrL) and infralimbic cortex (IL), and the frontal stem cell-rich subventricular zone (SVZ). Each of these regions is known to respond to environmental changes, so we hypothesized that development of each of these regions will show alterations with age as well as with adolescent environmental factors such as alcohol binge drinking.

Adolescent risk-taking and sensation-seeking behaviors include alcohol binge drinking and experimentation with other drugs (O'Malley et al., 1998; Wechsler et al., 1995). Human studies find chronic alcohol abuse decreases cerebral white matter and impairs executive function (de la Monte and Kril, 2014). Further, frontal white matter lipid profiles are altered in human alcohol use disorder (AUD) (de la Monte et al., 2018) and in adult rats by exposure to models of AUD (Yalcin et al., 2017). Mandyam and colleagues found that adult rat chronic intermittent ethanol exposure reduces medial PFC (mPFC) proliferation, differentiation, and survival of premyelinating OLs, and decreases MBP expression (Richardson et al., 2009; Kim et al., 2015) that reverses with protracted abstinence (Navarro and Mandyam, 2015). Human adolescent PFC brain regions have late-adolescent myelination growth trajectories related to performance maturation (Kwon et al., 2020), and adolescent binge drinking has been found to alter adolescent PFC growth trajectories (Pfefferbaum et al., 2018). Pascual and colleagues’ rat studies on adolescent binge-like ethanol administration were among the first to link proinflammatory cytokines and Toll-like receptors (TLRs) and alterations in expression of adult myelin proteins (Pascual et al., 2014). Their group extended these studies in adult wild-type and transgenic TLR4 knockout mice, finding using multiple methods including electron microscopy that chronic ethanol disrupts myelin membrane sheaths and increases proinflammatory cytokines and NG2, while decreasing OL protein expression that is markedly reduced in transgenic TLR4 knock out mice (Alfonso-Loeches et al., 2012). These studies were extended to adolescent alcohol exposure PFC-fmi myelin damage in rats (Vargas et al., 2014) as well as mice, finding losses in mPFC myelin and parvalbumin (Rice et al., 2019). Other studies investigating adolescent binge drinking find long-lasting changes in adult executive function, particularly behavioral flexibility, as well as PFC gene expression, physiology, and cell structure and function that are linked to neuroimmune gene induction (Crews et al., 2016; Crews et al., 2019). Our previous studies have found that adolescent intermittent ethanol (AIE) persistently decreases neurogenesis in the hippocampus and SVZ of adult rat brain (Ehlers et al., 2013a; Broadwater et al., 2014; Liu and Crews, 2017), which may be related to increased expression of proinflammatory cytokines and other innate immune signaling molecules (Vetreno and Crews, 2012). This study extends these studies to determine if AIE induces persistent changes in OPCs and OLs.

## Materials and Methods

### Animals and AIE Exposure

**FIGURE 1:**
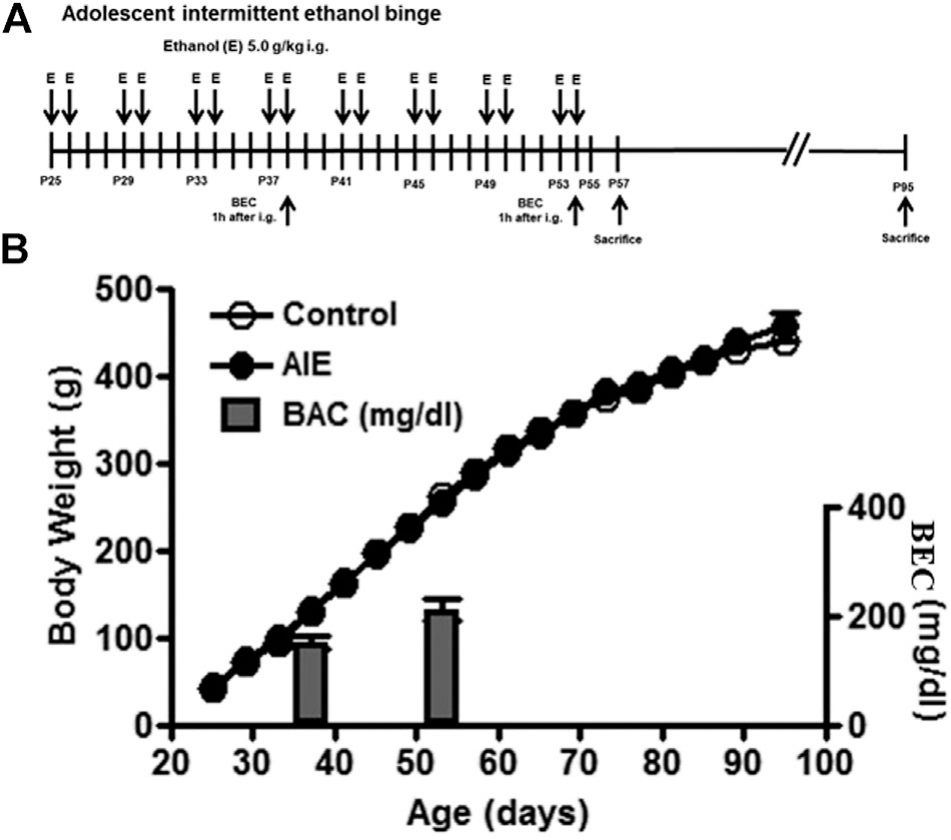
Experimental design, body weights and blood ethanol concentration (BEC). **(A)** The timeline of the experimental design. Adolescent intermittent ethanol (AIE) started at postnatal day (P)25. Adolescent animals were intermittently administered with either water or ethanol (5 g/kg, 25% ethanol w/v, intragastric) with 2 days on and 2 days off during adolescence (P25–P54). **(B)** Body weight was measured every four days during the procedure. There was no difference in the mean body weight between control and ethanol groups from P25 to P95. BEC was measured 1 h after treating with ethanol (5 g/kg, intragastric) at P38 and P54.

Timed-pregnant Wistar rats were ordered from Harlan Laboratories, Inc (Indianapolis, IN, USA) under a protocol approved by the Institutional Animal Care and Use Committee at the University of North Carolina at Chapel Hill. All animals were maintained at 22°C under a 12:12-h light/dark cycle with free access to food and water. Timed-pregnant dams at embryonic day 17 were allowed to acclimate to our vivarium. On the day following birth (postnatal day [P]1), litters were culled to 10 pups. On weaning at P21, male offspring, no more than one subject from each litter, were assigned to a single experimental condition to minimize the impact of litter variables. Rats were pair-housed with a same-sex, same-age non-littermate and then body weight match assigned to two experimental groups: control and adolescent intermittent ethanol (AIE) exposure, as used by the Neurobiology of Adolescent Drinking in Adulthood (NADIA) consortium (Crews et al., 2019; Crews et al., 2016). We employed intragastric (i.g.) water or ethanol administration (25% ethanol w/v, i. g. 10 ml/kg). We (Walter et al., 2017) and others (Arantes-Rodrigues et al., 2012) have established that intragastric water is not stressful when done properly. Animal welfare recommends this route for the volumes needed (Diehl et al., 2001; Turner et al., 2012). The AIE group was exposed intermittently (i.e., 2 days on, 2 days off) to ethanol during adolescence (P25-P54); the control group was administered the same volume of water as described previously (Figure 1A Liu and Crews, 2015; Liu and Crews, 2017). Control and AIE groups initially each had 16 (for IHC) or 18 (for RT-PCR) rats, with half being sacrificed on P57 (late adolescence) shortly after treatment ended and half, i.e., 8 (for IHC) or 9 (for RT-PCR) controls and 8 or 9 AIE, sacrificed on P95 (adulthood), 41 days following treatment. Tail blood samples were collected 1 h after ethanol treatment at P38 and P54 in the AIE groups. Blood ethanol concentrations (BECs) were measured using a GM7 Analyzer (Analox, London, UK). BEC ranged from 150 to 250 mg/dl (Figure 1B). Control (water) and AIE (ethanol 5 gm/kg) were dosed on a 2 days on/2 days off schedule from P38 to P54. There were no further treatments following the P54 ethanol exposure until sacrifice on P57 or P95.

### Animal Tissue Collection, Preparation and Immunohistochemistry

Rats were deeply anesthetized with an overdose of sodium pentobarbital, and transcardially perfused with 0.1 M phosphate-buffered saline (PBS, pH 7.4) followed by 4% paraformaldehyde (in 0.1 M phosphate buffer, pH 7.4). Brains were removed and post-fixed for 24 h in 4% paraformaldehyde at 4 °C. Coronal sections were obtained at a thickness of 40 μm in a 1:12 series after cryoprotection with 20 and 30% sucrose. Every 12th section was used for each immunohistochemical experiment.

For all antigens (Supplementary Table S1), sections were incubated in 0.6% H_2_O_2_ for 30 min to remove endogenous peroxidase activity, and blocked in 5% goat serum or rabbit serum (0.2% Triton X-100) for 1 h at room temperature. All primary antibodies were used at different dilutions (Supplementary Table S1) overnight at 4°C. On the second day, sections were rinsed in PBS and incubated with biotinylated secondary goat anti-rabbit or anti-mouse or rabbit anti-goat antibody (1:200, Vector Laboratories, Burlingame, CA, USA) for 1 h at room temperature. Subsequently, avidin-biotin-peroxidase complex (ABC Elite Kit, Vector Laboratories) was added for 1 h at room temperature. The positive expression was visualized using DAB (nickel-enhanced diaminobenzidine).

### Quantification for Immunohistochemistry

**FIGURE 2:**
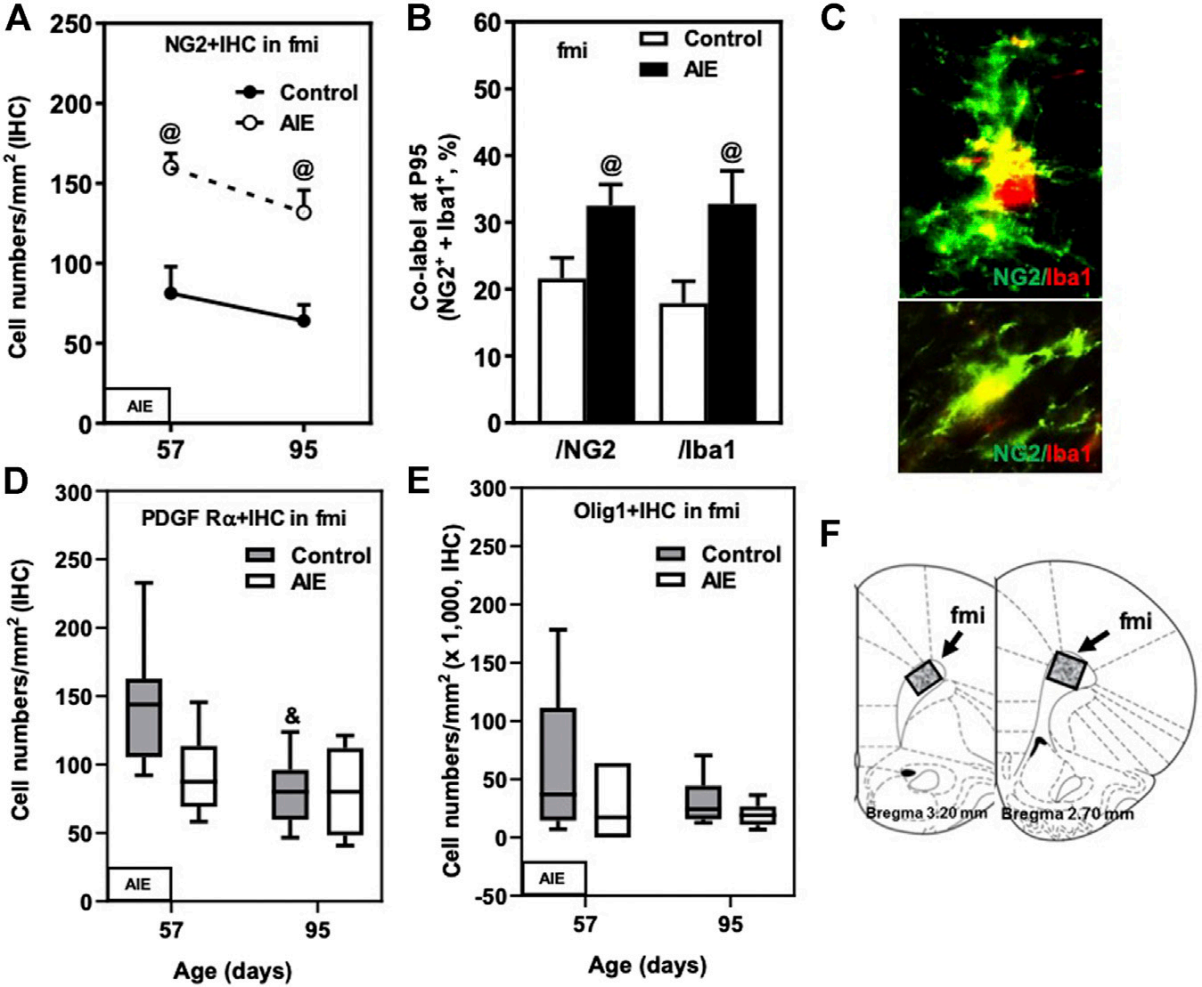
Effects of age and AIE on OL markers in frontal cortical white matter, the forceps minor of the corpus callosum (fmi). **(A)** NG2+IHC expression in the AIE group was higher at P57 (97%) and P95 (106%) compared with controls. **(B)** AIE exposure resulted in an increase of co-localization of NG2+/Iba1+ cells in NG2+ cells as well as in Iba1+ cells at P95 compared with controls. 50–100 NG2+ or Iba1+ positive cells per sample were analyzed for co-localization with Iba1+ or NG2+ cells. The percentage of co-localization in NG2+ or Iba1+ positive cells was calculated. **(C)** Photomicrographs of co-labeled images in the fmi, NG2+ (green) and Iba1+ (red), image 40×. **(D)** PDGF Rα+IHC expression showed maturational decline in controls from P57 to P95. **(E)** AIE exposure did not affect Olig1+IHC expression. **(F)** The fmi region of male rat brain was quantified by the histological method. The positive cells of oligodendrocyte progenitor and myelin markers of shaded areas were counted. Rat atlas panels were reprinted from Paxinos and Watson (2014). Data were expressed as the numbers of NG2+ **(A)**, PDGF Rα+ **(D)**, and Olig1+IHC **(E)** positive cells. Each point is mean ± SEM per mm^2^; *n* = 7–8/group.

A modified stereology method was used as described previously (Liu and Crews, 2017; Kuhn et al., 1997; Crews et al., 2006). Bioquant Nova Advanced Image Analysis (R&M Biometric, Nashville, TN) was used for image capture and analysis (Crews et al., 2004). Images were captured with an Olympus BX50 Microscope and Sony DXC-390 video camera linked to a computer. For the counting of NG2+, PGDFRα+, Olig1+ (in fmi and PFC), Olig2+, MAG+, MOG+ and CNPase + IHC, positive cells were counted using profile counting in the regions of interest and expressed as cells/mm^2^ with both sides of 3-5 sections per animal, and the average value per mm^2^ was used. For Olig1+ (in SVZ), MBP+ and PLP + IHC, pixel densities were measured for the outlined area (pixels/mm^2^). Rat brain coronal sections identify regions assessed: the forceps minor of the corpus callosum (fmi, Figure 2F), prelimbic (PrL) and infralimbic (IL) cortex (Figure 4H, bregma from 3.20 to 2.20 mm) and the SVZ (Figure 6G), bregma from 1.20 to 0.70 mm (Puelles et al., 2013). A modified stereology method measured positive immunoreactivities in a series of three 50 μm boxes along the length of SVZ as previously described (Kuhn et al., 1997; Crews et al., 2006; Liu and Crews, 2017). For overlay analysis, images of NG2+ andIba1+ positive cells and their overlay cells within the fmi, PrL, and SVZ regions were digitally captured using an NIS-Element AR by Nikon. Within delineated areas, 50–100 Iba1+ or NG2+ positive cells per sample were analyzed for overlaying with NG2+ or Iba1+. The percentages of overlay in Iba1+ or NG2+ position cells were calculated.

### PFC RNA Extraction and Reverse Transcription PCR (RT-PCR)

Total RNA was extracted from frozen PFC tissue samples from adult (P95, *n* = 9/each group) control- and AIE-treated subjects by homogenization in TRI reagent (Sigma-Aldrich, St. Louis, MO) following the single-step method of RNA isolation (Chomczynski and Sacchi, 2006). RNA quality and concentration was determined using a NanoDrop 1,000 (ThermoFisher Scientific, Austin, TX). Total mRNA was transcribed into complementary DNA by reverse transcriptase as previously described (Vetreno and Crews, 2018; Vetreno et al., 2018; Liu et al., 2020). RT-PCR reactions were run on a BioRad CFX system (BioRad, Hercules, CA). SYBER Green PCR Master Mix (Life Technologies, Carlsbad, CA) was used for RT-PCR. RT-PCR was run with an initial activation for 10 min at 95°C, followed by 40 cycles of denaturation (95 °C, 15 s), annealing/extension (57–58 °C, 1 min), and melt curve. The primer sequences are presented in Supplementary Table S2. Differences in primer expression between groups are expressed as cycle time (Ct) values normalized with β-actin, and relative differences between groups calculated and expressed as the percent difference relative to controls.

### Statistical Analysis

All data was analyzed using SPSS (IBM SPSS Statistics 22) and initially assessed using the Shapiro-Wilk test to determine normality of data distribution. Normal distributed data was assessed with a 2 × 2 ANOVA with follow-up Tukey's post-hoc tests and presented as mean ± SEM. Data with non-normal distribution was assessed using the non-parametric Kruskal-Wallis test followed by post-hoc pairwise multiple comparisons adjusted by the Bonferroni test and are presented as median ± IQR (BOLD TEXT in Tables). For the normal distribution data, the percentage of maturational decline between P57 and P95 with marker + IHC expression was (P57-P95)/P57*100; and the percentage of maturational increase between P57 and P95 with marker + IHC expression was (P95-P57)/P57*100. The percentage of AIE-induced decrease was (Control-AIE)/Control*100; and percentage of AIE-induced increase was (AIE-Control)/Control*100.

## Results

We initiated treatments at P25, an early pre-pubertal adolescent age, when controls (water) and AIE groups (5 g/kg, i. g. 16 doses over 30 days, Figure1A) weighed 44 ± 1 g and 46 ± 1 g, respectively. Body weight was recorded every four days and increased in parallel through puberty with control and AIE groups being 263 ± 4 and 258 ± 5 g, respectively, at P54 after the last exposure to ethanol (Figure 1B). AIE BECs were measured at P38 and at P54, being 152 ± 11 and 212 ± 19 mg/dl, respectively (Figure 1B). Body weight at P95 was 446 ± 10 for controls and 454 ± 9 for AIE. OPC and OL markers were assessed at P57 (late adolescence) and P95 (adulthood). Control and AIE groups showed significant changes in weight with age, but not between groups.

### Frontal White Matter Maturation in the Corpus Callosum Forceps Minor and the Impact of AIE

Oligodendrocyte precursor cells (OPCs) express the proteoglycan NG2 and the PDGF receptor α (PDGFRα) as well as Olig1 and Olig2. We compared P57, a post-pubertal adolescent age, to a P95 mature adult rat and found abundant expression of all of these OPC markers in the fmi (Figure 2; Table 1). NG2+IHC cells did not change across aging in fmi from P57 to P95 (Figure 2A; Table 1). In contrast, PDGFRα+IHC cells were more abundant at P57 and showed a maturational decline between P57 and P95 in control group (Figure 2D; Table 1). At P95, the density of NG2+IHC and PDGFRα+IHC cells were similar, consistent with adult OPC not declining with age. We did not find significant maturational changes in the expression of Olig1+ (Figure 2E; Table 1), Olig2+ (Table 1), or CNPase + IHC (Table 1) in the fmi. AIE exposure significantly increased the expression of the OPC marker NG2+IHC in the fmi across ages, being about double at P57 (*p* < 0.05) and P95 (*p* < 0.05, Figure 2A and Table 1). Interestingly, AIE increased co-localization of NG2+ cells with Iba1+ positive cells. NG2+/Iba1+ co-localization in control fmi was 22 ± 3% in total NG2+ positive cells and 18 ± 3% in total Iba1+ positive cells at P95, with AIE animals having much more co-localization of cells (33 ± 3% in total NG2+ and 33 ± 5% in total Iba1+ positive cells, *p* < 0.05) (Figures 2B,C). AIE exposure decreased PDGFRα+IHC cells at P57; however, there was no maturational decline in AIE PDGFRα+IHC cells paralleling the control maturational loss at P95, resulting in adult PDGFRα+IHC cells at P95 that were similar to controls (Figure 2D; Table 1). We did not find significant maturational or AIE-induced changes in the expression of Olig1+ (Figure 2E; Table 1), Olig2+ (Table 1), or CNPase + IHC (Table 1) in the fmi. These findings suggest that NG2+ progenitors in fmi continue to change with maturation into adulthood and that AIE exposure alters NG2 cell expression and association with Iba1+ microglial cells.

**TABLE 1:** Frontal cortical white matter, rat forceps minor of the corpus callosum (fmi) oligodendrogenesis markers at P57 and P95: Effects of adolescent intermittent ethanol (AIE) exposure.

Marker	Postnatal day 57		Postnatal day 95	
Group	Control	AIE	Control	AIE
NG2 (cell No/mm^2^)	81 ± 17	160 ± 8.9^@^↑	64 ± 9.9	132 ± 13^@^↑
**PGDF Ra (cell No/mm** ^**2**^ **)**	**144 (26)**	**87 (33)**	**80 (28)** ^a^ ^↓^	**80 (52)**
**Olig1 (cell No/mm** ^**2**^ **)**	**37 (58)**	**23 (53)**	**24 (17)**	**19 (13)**
Olig2 (cell No/mm^2^, ×10^3^)	6.5 ± 0.6	5.7 ± 0.5	3.8 ± 0.4^b^ ^↓^	4.5 ± 0.5
CNPase (cell No/mm^2^,×10^3^)	1.2 ± 0.1	1.2 ± 0.2	1.1 ± 0.1	1.0 ± 0.2
**MBP (pixels/mm** ^**2**^ **, ×10** ^**3**^ **)**	**10 (7)**	**17 (12)**	**11 (6)**	**21 (14)** ^**@**^ **↑**
**PLP (pixels/mm** ^**2**^ **, ×10** ^**3**^ **)**	**14 (7)**	**7 (4)**	**5 (2)** ^c^ ^↓^	**9 (3)**
**MAG (cell No/mm** ^**2**^ **, ×10** ^**3**^ **)**	**2 (0.2)**	**2 (0.3)**	**2 (0.7)**	**2 (0.9)**
MOG (cell No/mm^2^, ×10^3^)	1.2 ± 0.1	1.5 ± 0.1	1.0 ± 0.1	0.7 ± 0.1^c^ ^↓^

Data are expressed as the numbers of NG2+, PDGF Rα+, Olig1+, Olig2+, CNPase+, MAG+ and MOG + IHC positive cells, and the pixels of MBP+ and PLP + IHC expression. Normality of distribution was assessed using the Shapiro-Wilk test. Data with normal distribution was assessed using a 2 × 2 ANOVA with Tukey's HSD post-hoc tests when appropriate and presented as mean ± SEM. Data that was not normally distributed and assessed using the non-parametric Kruskal–Wallis test are indicated in BOLD.

^@^ *p* < 0.05 compares with control group at the same age.

a *p* < 0.05.

b *p* < 0.01.

c *p* < 0.001 compared with either control or AIE group at P57 separately. Arrow “↑” represents increase; “↓” represents decease. Data are presented as mean ± SEM per mm^2^; *n* = 7–8/group.

**FIGURE 3:**
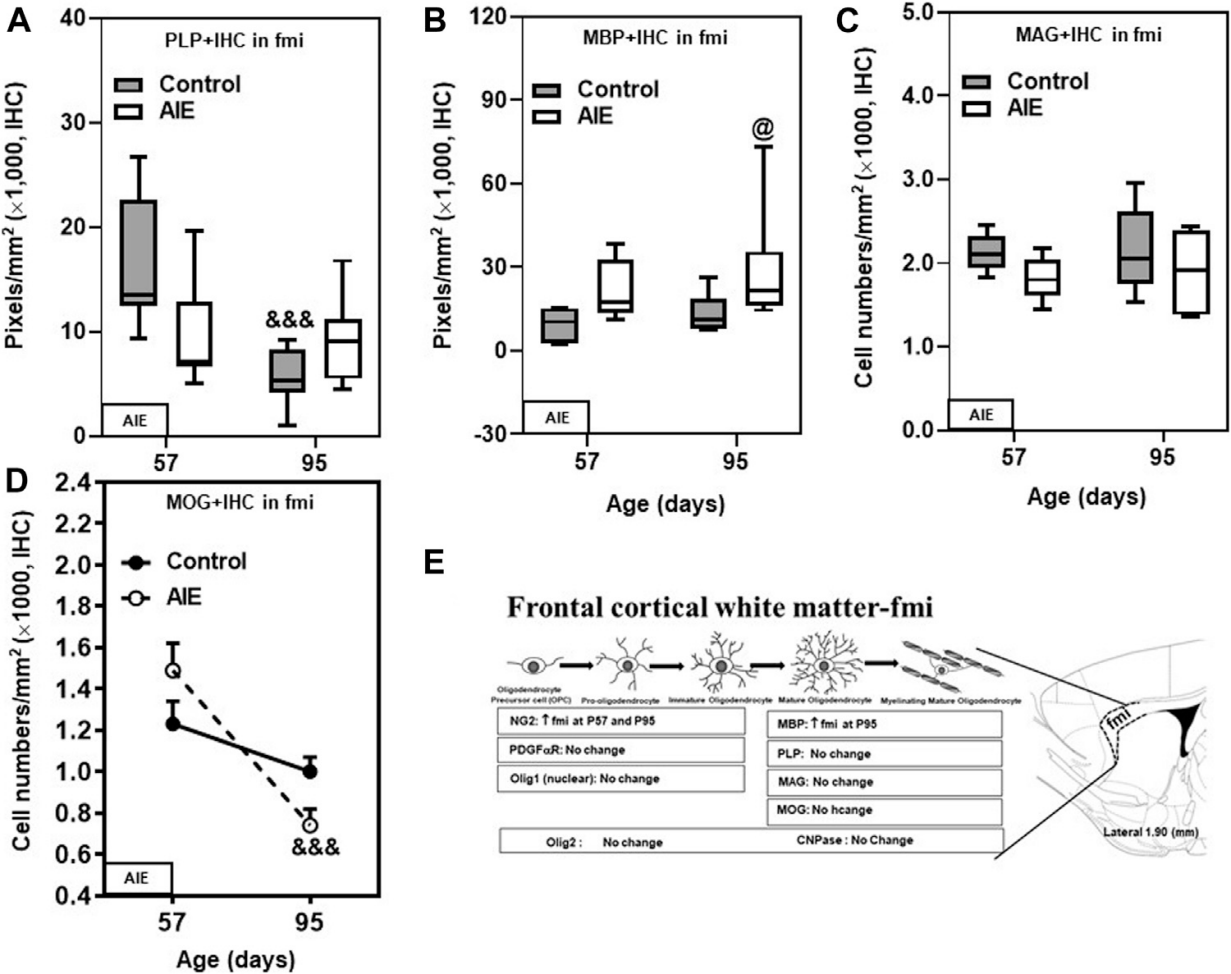
Effects of age and AIE on OL markers in frontal cortical white matter, the forceps minor of the corpus callosum (fmi). **(A)** PLP + IHC expression showed maturational decline from P57 to P95 in controls, but not in the AIE group. **(B)** AIE exposure increased MBP + IHC expression at P95 compared with controls. **(C)** AIE exposure did not affect MAG + IHC expression. **(D)** MOG + IHC expression in the AIE group showed maturational decline from P57 to P95 (66%). **(E)** Schematic diagram representing the developmental stages of oligodendrogenesis in the fmi. The specific markers that are expressed in distinct stages are indicated below, Arrow “↑” represents increase; “↓” represents decease; “at P57” and “at P95” represents at postnatal days 57 and 95 respectively; “no change” means that AIE did not affect the markers’ expression. Rat atlas panel of the right side reprinted from Paxinos and Watson (2014). Data were expressed as the pixels of PLP+ **(A)** and MBP + IHC **(B)** and numbers of MAG+ **(C)** and MOG + IHC **(D)** positive cells. Each point is mean ± SEM per mm^2^; *n* = 7–8/group.

The major mature myelin proteins, which include the hydrophobic transmembrane proteolipid protein (PLP), extrinsic and hydrophilic myelin basic protein (MBP), myelin-associated glycoprotein (MAG), and myelin oligodendrocyte glycoprotein (MOG)**,** contribute to the structure of myelin lipid membranes. Among these mature markers, only PLP + IHC expression changed with maturation. The decline in PLP + IHC expression between P57 and P95 in fmi (*p* < 0.001, Figure 3A and Table 1). Maturation of controls to P95 resulted in a marked loss of PLP + IHC to a level below AIE P57 levels, and AIE did not show a developmental decrease, resulting in AIE PLP + IHC being greater than controls at P95 (Figure 3A; Table 1). MBP + IHC did not change with age; however, AIE exposure doubled MBP + IHC expression in the fmi at both P57 and P95 (*p* < 0.05, Figure 3B and Table 1). However, AIE exposure slightly decreased mature OL marker MAG + IHC in the fmi at P57 (Figure 3C; Table 1). Interestingly, AIE exposure decreased expression of another mature OL marker, MOG + IHC, in the fmi at P95 (27%, Figure 3D and Table 1). MOG + IHC expression in the AIE group showed a maturational decline between P57 and P95 (66%, *p* < 0.001, Figure 3D and Table 1). These findings suggest these mature myelin proteins have different developmental trajectories in fmi and that AIE alters the trajectory in complex ways.

### Frontal Gray Matter Maturation in the Prelimbic and Infralimbic Cortex: Impact of Adolescent Intermittent Ethanol Exposure

**FIGURE 4:**
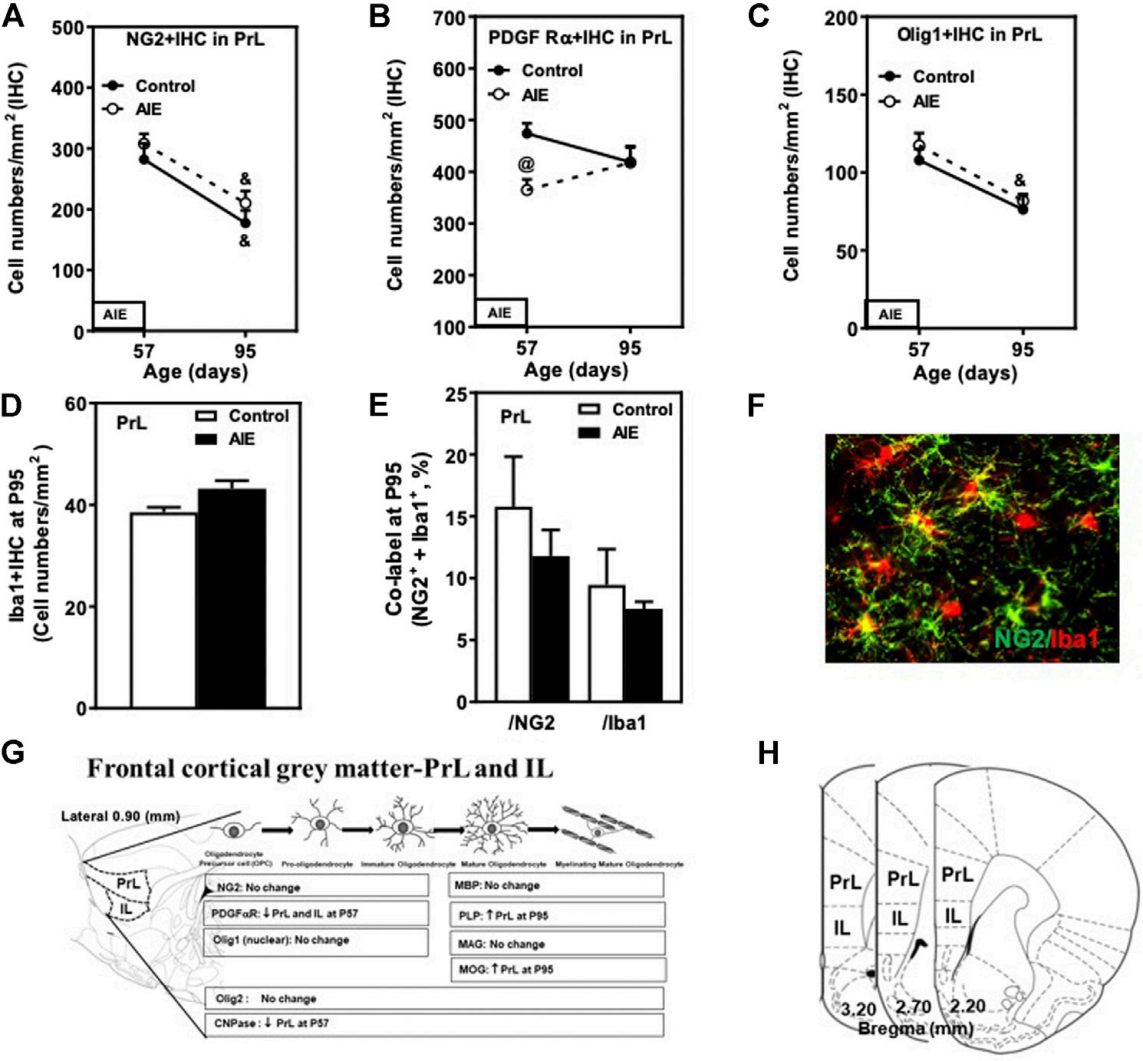
Effects of age and AIE on NG2 and OPC markers in the frontal gray matter, prelimbic cortex (PrL). **(A)** NG2+IHC expression showed maturational decline in both controls (48%) and the AIE group (36%). **(B)** AIE exposure decreased 34% of PDGF Rα+IHC at P57. **(C)** Olig1+IHC expression showed maturational decline in both controls (31%) and the AIE group (30%). **(D)** AIE exposure resulted in an 16% increase in Iba1+IHC expression (*p* = 0.056) at P95 compared with controls. **(E)** AIE exposure did not affect co-localization of NG2+/Iba1+ cells in either NG2+ cells or Iba1+ cells at P95 compared with controls. 50–100 NG2+ or Iba1+ positive cells per sample were analyzed for co-localization with Iba1+ or NG2+ cells. The percentage of co-localization in NG2+ or Iba1+ positive cells was calculated. **(F)** Photomicrographs of co-labeled images in the PrL, NG2+ (green) and Iba1+ (red), image 20×. **(G)** Schematic diagram representing the developmental stages of oligodendrogenesis in the frontal cortical gray matter-prelimbic (PrL) and infralimbic (IL) cortex. The specific markers that are expressed in distinct stages are indicated below, Arrow “↑” represents increase; “↓” decease; “at P57” and “at P95” represents at postnatal days 57 and 95 respectively; “no change” means that AIE did not affect the markers’ expression. Rat atlas panel of the left side reprinted from Paxinos and Watson (2014). **(H)** The frontal cortical gray matter and the prelimbic (PrL) and infralimbic (IL) cortex of male rat brain were used by the histological method. The positive cells of oligodendrocyte progenitor and myelin markers of shaded areas were counted. Rat atlas panels were reprinted from Paxinos and Watson (2014). Data were expressed as the numbers of NG2+ **(A)**, PDGF Rα+ **(B)**, Olig1+ **(C)** and Iba1+IHC **(D)** positive cells. Each point is mean ± SEM per mm^2^; *n* = 7–8/group.

Prefrontal cortical gray matter maturation includes increasing myelination. NG2+ and PDGFRα+IHC are highly expressed at P57 in both PrL (Figures 4A,B; Table 2) and IL (Table 2). NG2+ and Olig1+IHC showed declines between P57 and P95 in both control and AIE groups (Figures 4A,C; Table 2). The maturational decrease of 40–50% in NG2+IHC cells and a lack of change in PDGFRα+IHC in both PFC gray matter regions contrasts with that found in nearby white matter fmi. In PrL and IL PFC gray matter, AIE exposure did not affect NG2+IHC (PrL and IL; Figure 4A and Table 2). PDGFRα+IHC expression was reduced by AIE in both the PrL (*p* < 0.05, Figure 4B and Table 2) and the IL (*p* < 0.05, Table 2) at P57, but not at P95 after AIE exposure. Interestingly, AIE exposure induced a 12% increase of Iba1+IHC expression in PrL at P95 (*p =* 0.056, Figure 4D), but did not change NG2+ and Iba1+ co-localization (Figures 4E,F). Olig1+IHC expression in the PrL (Figure 4C; Table 2) and IL (Table 2) showed the maturational decrease in controls and AIE across both the PrL and the IL. These findings suggest PFC gray matter OPCs show maturational declines between P57 and P95 that are not markedly altered by AIE exposure.

**TABLE 2:** Effects of adolescent intermittent ethanol (AIE, 5 g/kg, i.g., 2 days on/2 days off) exposure on oligodendrogenesis markers in the frontal cortical gray matter, prelimbic (PrL) and infralimbic (IL) cortex of male rat brain at P57 and P95.

Marker	Subregion	Postnatal day 57		Postnatal day 95	
**Group**		Control	AIE	Control	AIE
**NG2 (Cells/mm** ^**2**^ **)**	PrL	282 ± 26	308 ± 14	177 ± 21^a^ ^↓^	210 ± 20^a^ ^↓^
	IL	314 ± 32	317 ± 58	165 ± 16^b^ ^↓^	202 ± 16
**PGDF rα (Cells/mm** ^**2**^ **)**	PrL	474 ± 23	365 ± 21^@^↓	419 ± 31	418 ± 30
	**IL**	**411 (129)**	**276 (55)** ^**@**^ **↓**	**299 (67)**	**369 (139)**
**Olig1 (Cells/mm** ^**2**^ **)**	PrL	108 ± 7.2	117 ± 8.0	76 ± 10	82 ± 3.7^a^ ^↓^
	**IL**	**96 (22)**	**113(29)**	**72 (29)** ^a^ ^↓^	**78 (4)** ^a^ ^↓^
**Olig2 (Cells/mm** ^**2**^ **, ×10** ^**3**^ **)**	PrL	1.0 ± 0.0	0.9 ± 0.1	0.8 ± 0.0^a^ ^↓^	0.9 ± 0.0
	IL	1.1 ± 0.1	1.0 ± 0.1	0.8 ± 0.1^a^ ^↓^	0.9 ± 0.0
**CNPase (Cells/mm** ^**2**^ **)**	**PrL**	**200 (70)**	**91 (37)** ^**@**^ **↓**	**89 (14)** ^b^ ^↓^	**154 (64)**
	IL	170 ± 18	126 ± 19	111 ± 17	163 ± 20
**MBP (Pixels/mm** ^**2**^ **, ×10** ^**3**^ **)**	**PrL**	**15 (7)**	**14 (11)**	**16 (10)**	**19 (13)**
	**IL**	**9 (4)**	**5 (3)**	**15 (9)**	**9 (3)**
**PLP (Pixels/mm** ^**2**^ **, ×10** ^**3**^ **)**	**PrL**	**4 (3)**	**4 (3)**	**11 (4)**	**31(15)** ^b^ ^↑^
	**IL**	**3 (2)**	**2 (2)**	**8 (4)** ^a^ ^↑^	**10 (7)** ^b^ ^↑^
**MAG (Cells/mm** ^**2**^ **)**	PrL	346 ± 23	254 ± 13	320 ± 36	403 ± 24^b^ ^↑^
	**IL**	**269 (51)**	**225 (28)**	**324 (88)**	**320 (129)** ^a^ ^↑^
**MOG (Cells/mm** ^**2**^ **)**	PrL	380 ± 33	261 ± 18	189 ± 13^c^ ^↓^	275 ± 19^@^↑
	**IL**	**362 (153)**	**273 (125)**	**125 (66)** ^c^ ^↓^	**168 (42)**

Data are expressed as the numbers of NG2+, PDGF Rα+, Olig1+, Olig2+, CNPase+, MAG+ and MOG + IHC positive cells, and the pixels of MBP+ and PLP + IHC expression. Normality of distribution was assessed using the Shapiro-Wilk test. Data with normal distribution was assessed using a 2 × 2 ANOVA with Tukey’s HSD post-hoc tests when appropriate and presented as mean ± SEM. Data that was not normally distributed and assessed using the non-parametric Kruskal-Wallis test are indicated in BOLD.

^@^ *p* < 0.05 compares with control group at the same age.

a *p* < 0.05.

b *p* < 0.01.

c *p* < 0.001. compared with either controls or AIE group at P57, separately. Arrow “↑” represents increase; “↓” represents decease. Data are presented as mean ± SEM per mm^2^; *n* = 7–8/group.

**FIGURE 5:**
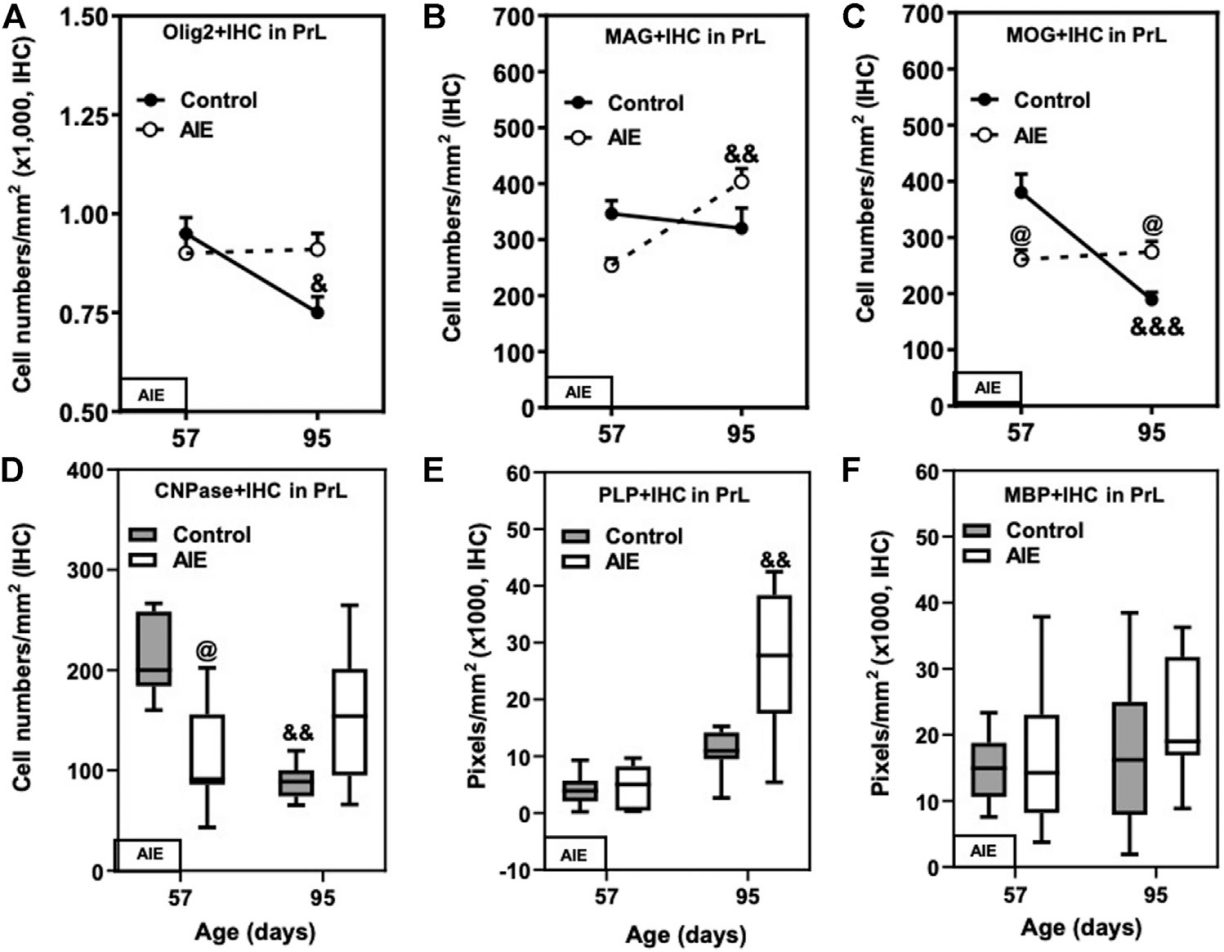
Effects of age and AIE on OL markers in the frontal cortical gray matter prelimbic cortex (PrL). **(A)** Olig2+IHC expression in the AIE group was greater at P95 than in controls (21%). Olig2+IHC expression showed maturational decline in controls (20%), but not in the AIE group. **(B)** MAG + IHC expression in the AIE group showed maturational augmentation from P57 to P95 (59%). **(C)** AIE exposure decreased MOG + IHC expression at P57 (31%); however, MOG + IHC expression in AIE group was greater than in controls (53%). MOG + IHC expression in controls showed maturational decline from P57 to P95 (48%). **(D)** AIE exposure remarkably decreased CNPase + IHC expression at P57 compared with controls; however, CNPase + IHC expression in the AIE group was greater than controls at P95 because of significant maturational decline of controls between P57 and P95. **(E)** PLP + IHC expression at P95 was higher in the AIE group than controls, and there was maturation augmentation in controls and the AIE group from P57 to P95. **(F)** AIE exposure did not affect MBP + IHC expression compared with controls. Data were expressed as numbers of Olig2+ **(A)**, MAG+ **(B)**, MOG + IHC **(C)** and CNPase+ **(D)**, positive cells and the pixels of PLP+ **(E)** and MBP+ **(F)**. Each point is mean ± SEM per mm^2^; *n* = 7–8/group.

Although myelination of PFC in rat continues to increase in brain from late adolescence into adulthood (Mengler et al., 2014), in PFC, PrL and IL gray matter expression of Olig2+, CNPase+, and MOG + IHC decreased between P57 and P95 while PLP + IHC increased (Figures 5A,C,D,E; Table 2). There was a maturational decline between P57 and P95 of the OL lineage marker Olig2+IHC in the control PrL (20%, *p* < 0.05, Figure 5A and Table 2) and IL (23%, *p* < 0.05, Table 2). AIE exposure also significantly decreased CNPase + IHC expression in the PrL at P57; however, AIE groups recovered over time such that at P95, CNPase + IHC expression was greater in AIE than controls (Figure 5D; Table 2). PLP + IHC expression was uniquely increased with age among these mature myelin markers in both PFC gray matter regions from P57 to P95, and this was remarkably augmented by AIE, i.e., in PrL (Figure 5E; Table 2) and in IL (Table 2). MAG + IHC expression showed little change with age in control group, whereas it decreased just after AIE exposure (at P57) in the PrL and IL, and recovered by P95 (Figure 5B; Table 2). There was a maturational increase in MAG + IHC expression in the PrL (*p* < 0.01) and IL (*p* < 0.05) of AIE. There was a maturational decline in MOG + IHC expression in the PrL of controls (48%, *p* < 0.001, Figure 5C and Table 2) with AIE showing a pattern similar to MAG + IHC of AIE altering maturational changes, resulting in P57 decreases and P95 increases in expression. MBP + IHC was not changed in PrL and IL by age or AIE (Figure 5F; Table 2). PLP and MBP are highly expressed proteins that do not decline between P57 and P95 and could reflect the increases in myelin membranes and lipids. These findings indicate prefrontal gray matter has maturational changes in OLs and OPCs between late adolescence and mature adulthood that are altered by AIE.

### Subventricular Zone (SVZ) Maturation and the Impact of AIE

**FIGURE 6:**
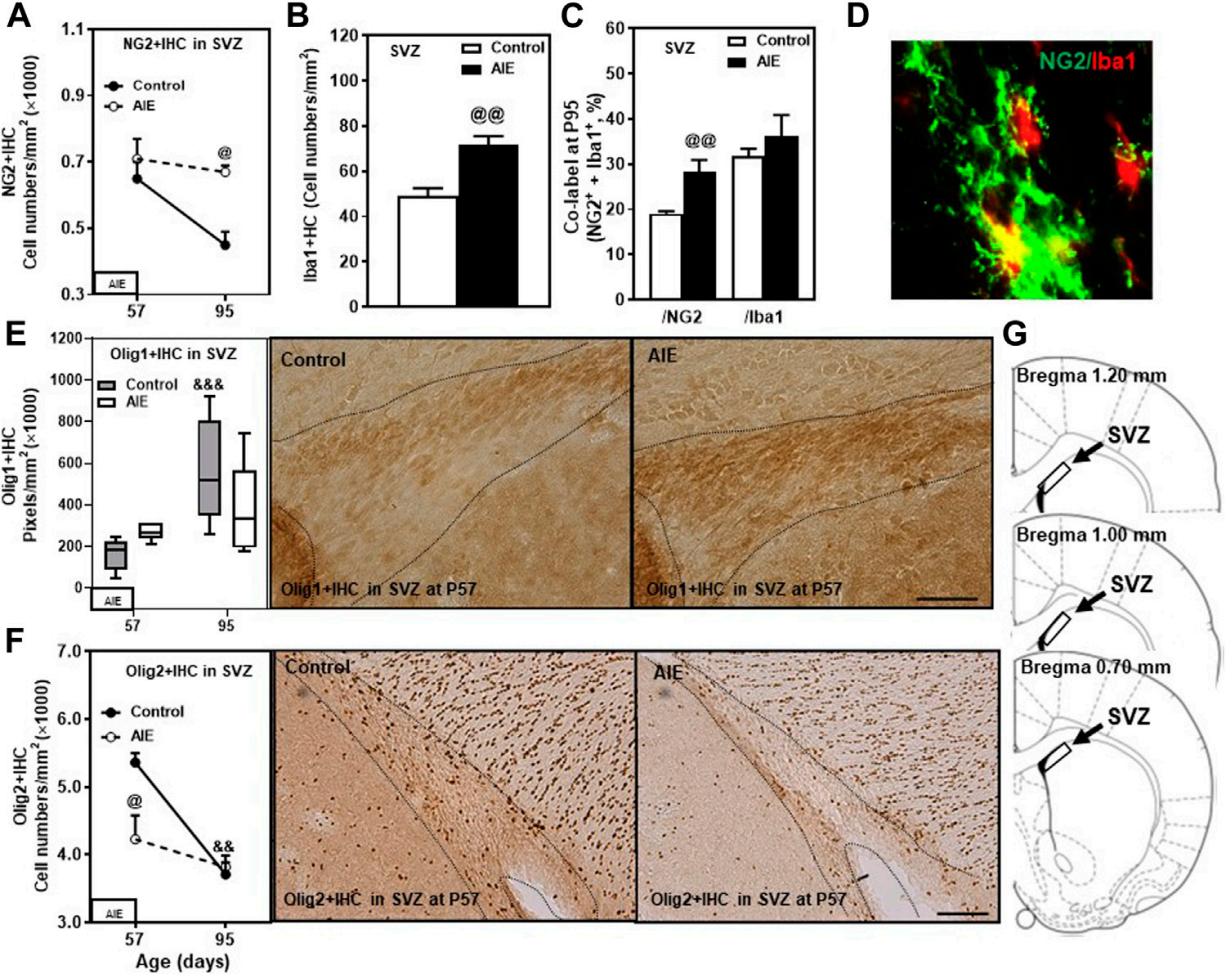
Effects of age and AIE on OPC-OL markers in the subventricular zone (SVZ). **(A)** NG2+IHC expression was higher in the AIE group than controls at P95 (50%). NG2+IHC expression showed maturational decline in controls (32%). **(B)** AIE exposure resulted in an increase of Iba1+IHC expression (47%) compared with controls at P95. **(C)** AIE exposure resulted in an increase of co-localization of NG2+/Iba1+ cells in total NG2+ cells (28%) compared with controls at P95.50–100 NG2+ or Iba1+ positive cells per sample were analyzed for co-localization with Iba1+ or NG2+ cells. The percentage of co-localization in NG2+ or Iba1+ positive cells was calculated**. (D)** Photomicrographs of co-labeled images in the SVZ, NG2+ (green) and Iba1+ (red), image 40×. **(E)** Left side: Olig1+IHC expression showed maturational augmentation in controls from P57 to P95. Right panel: Olig1+IHC expression in the SVZ (Immunohistochemical staining, Bar scale = 25 µm). **(F)** Left side: AIE exposure decreased Olig2+IHC expression at P57 (23%) but not at P95. Olig2+IHC expression showed maturational decline in control group from P57 to P95 (31%). Right panel: Olig2+IHC expression in SVZ (Immunohistochemical staining, Bar scale = 50 µm). **(G)** The subventricular zone of male rat brain was used by the histological method. The positive cells of oligodendrocyte progenitor markers of frame areas were counted. Rat atlas panels were reprinted from Paxinos and Watson (2014). Data were expressed as the numbers of NG2+ **(A)**, Iba1+ **(B)** and Olig2+IHC **(F)** positive cells, and the pixels of Olig1+IHC **(E)**. Each point is mean ± SEM per mm^2^; *n* = 7–8/group.

The rat SVZ of the lateral ventricles is a unique region of adolescent and adult oligodendrogenesis and neurogenesis (Ortega et al., 2013). OPCs in the SVZ express Olig2 and migrate to form OL in the corpus callosum and other white matter regions (Gonzalez-Perez and Alvarez-Buylla, 2011). In the present study, we determined transcription factors Olig1 and Olig2 as well as OPC marker NG2 and microglial marker Iba1 in SVZ (see Figure 6). NG2 cells in the SVZ become OLs, but neurons do not (Komitova et al., 2009). In SVZ, we found a much higher NG2+IHC cell density (Figure 6A) compared to PFC white matter (fmi) and gray matter (PrL, IL), although we observed similar levels of Olig1+ and Olig2+IHC expression. Each of these changed from P57 to P95 (Figures 6A,E,F). NG2+IHC expression from P57 to P95 declined 32% (Figure 6A) and Olig2+IHC declined 31% (*p* < 0.01, Figure 6F), whereas OL Olig1+IHC increased (*p* < 0.001, Figure 6E). AIE blunted maturation of these markers as well as increasing the microglial marker Iba1. NG2+IHC cells were not altered at P57 just after AIE exposure ended, but AIE treatment blocked the maturational decline, resulting in a 50% increase in NG2+IHC cells in AIE at P95 (*p* < 0.05, Figure 6A). AIE exposure also resulted in a 47% increase (*p* < 0.01, Figure 6B) in Iba1+IHC cells at P95 and increased co-localization of NG2+/Iba1+ in total NG2+ positive cells (28%; *p* < 0.01, Figures 6C,D) in SVZ. AIE exposure increased Olig1+IHC and decreased Olig2+IHC at P57 just after AIE exposure ended, but by P95 they had returned to values similar to age-matched controls (Figures 6E,F). For Olig1+IHC, These finding are consistent with SVZ undergoing significant changes in OPCs and OLs during maturation from P57 to P95 as well as AIE causing long-lasting changes in SVZ NG2+IHC glia, OPC, Iba1+IHC microglia and the associations of NG2 with Iba1.

### OPC and OL PFC mRNA Expression

The levels of expression of OPC and OL mRNA were determined in a separate group of control- and AIE-treated subjects at P95 for comparisons with the protein immunohistochemistry. The PFC brain regional differences in cellular protein immunoreactivity were unexpected, and these studies determined mRNA expression from the entire PFC. The OPC marker NG2 mRNA did not change following AIE treatment (Table 3). MOG mRNA showed a trend to increase that was not statistically significant, which is consistent with the increases in MOG + IHC in PrL gray matter that are blunted when paired with decreases in fmi. MBP mRNA (Table 3) was not significantly changed for the entire PFC, consistent with our finding of increases in fmi white matter and decreases in IL gray matter MBP + IHC (Table 2). Similarly, we found no effect of AIE on CNPase mRNA in P95 AIE rats. These findings are consistent with our assertion that marked cellular and brain PFC subregions identified by immunohistochemical determinations are lost with entire PFC, OPC and OL mRNA assessments.

**TABLE 3:** Effects of adolescent intermittent ethanol (AIE, 5 g/kg, i.g., 2 days on/2 days off) exposure on oligodendrocyte mRNA gene expression in the prefrontal cortex of male rat brain at P95.

Gene	Postnatal day 95	
Group	Control	AIE
***NG2***	100 ± 7	97 ± 11
***CNPase***	100 ± 12	99 ± 13
***MBP***	100 ± 10	77 ± 10
***MOG***	100 ± 13	142 ± 21

Data are expressed as percentage of mRNA gene expression of *NG2*, *CNPase*, *MBP*, and *MOG* in the prefrontal cortex of male AIE-treated rats at P95, relative to age-matched controls. Data are presented as mean ± SEM; *n* = 9/group.

## Discussion

In this study, we investigated the expression of OPC markers NG2, PDGFRα, and transcription factors Olig1 and Olig2 as well as several OL proteins, including MBP, PLP, MAG, MOG, and CNPase in post-pubertal adolescent (P57) and adult (P95) rat frontal white matter (fmi), frontal gray matter (PrL, IL) and frontal neuro-oligogenesis subventricular zone (SVZ). We found the OPC markers NG2+, PDGFRα+ and Olig2+IHC, across frontal white matter, gray matter and SZV neurogenic zones. In the SVZ, neural stem cells give rise to OL cells as well as neurons (Alvarez-Buylla and Garcia-Verdugo, 2002; El Waly et al., 2014). OLs progress through a series of differentiation steps from OPCs to mature OL cells, which are able to produce myelin (Nishiyama et al., 2009; Fröhlich et al., 2011; Marques et al., 2016). In general, OPC and OL markers decreased between adolescence (P57) and adulthood (P95). However, these markers had different cellular density and the changes are not in parallel, consistent with NG2+IHC cells being OPC polydendrocytes and other forms of progenitor stem cells (Nishiyama et al., 2009). Although these factors make it difficult to clearly identify OPCs, in SVZ, a progenitor brain region in adolescent and adult rats, we found NG2+ and Olig2+IHC cells/mm^2^ declined by about one-third in density from P57 to P95. Similarly, in IL prefrontal gray matter, NG2+, PDGFRα+, Olig2+ and Olig1+IHC declined from P57 to P95. Interestingly in fmi, PDGFRα+ and Olig2+IHC declined, but not NG2+IHC. *In vitro* studies have shown that PDGF is a mitogen and survival factor for OPCs (Grinspan and Franceschini, 1995). Studies in PDGFRα reporter mice find OPCs continue to proliferate and generate myelin in adulthood, although proliferation rate varies with age and between gray and white matter, all of which impact the density of +IHC markers (Moyon et al., 2016). We assessed mature OL markers and found many markers changed with age, although sometimes in opposite directions. For example, PLP+ and MBP + IHC showed strikingly different patterns across the two ages studied, with PLP decreasing by more than half in fmi white matter but no change in MBP + IHC, whereas in PrL gray matter, PLP + IHC more than doubles from P57 to P95, and MBP + IHC does not change. In PFC gray matter, NG2+, Olig1+, Olig2+, CNPase+, and MOG + IHC all showed age-related decreases, but not parallel declines. The OL markers contribute different amounts of OL protein. Hydrophobic PLP and hydrophilic MBP are major myelin proteins accounting for 40 and 30% of total protein, respectively (Boison et al., 1995; Boggs, 2006; Aggarwal et al., 2011), with 4% CNPase (Braun et al., 1991; de Monasterio-Schrader et al., 2012) as well as MAG and MOG each about 1% of CNS myelin protein (de Monasterio-Schrader et al., 2012), possibly contributing to a lack of parallel changes. Studies assessing white matter through MRI or myelin lipid stains show robust developmental changes that likely impact markers’ cell density for OL differentially. Both PLP and MBP are abundantly expressed in OL, have multiple forms, and bind to each other as well as themselves, thereby stabilizing the predominantly lipid myelin membranes (Edwards et al., 1989; Harauz et al., 2004). Our finding of differential changes in PFC PLP and MBP is consistent with MRI findings of adolescent to adult maturation of PFC myelin diffusivity (Vetreno et al., 2016). These findings are consistent with maturation and age-related changes in OPC and OL markers reflecting ongoing remodeling at different rates in PFC gray matter, fmi white matter and neurogenic SVZ continuing from adolescence into adulthood.

In addition to age, we determined the impact of AIE, a model of adolescent binge drinking, just after exposure and after maturation to adulthood. AIE is studied by the Neurobiology of Adolescent Drinking in Adulthood (NADIA) consortium to test the hypothesis that adolescent binge drinking disrupts maturation to adulthood which contributes to persistent adult pathology independent of progression to adult alcohol use disorder. The NADIA has found long-lasting AIE-induced adult increased alcohol drinking, increased anxiety (particularly social anxiety), increased impulsivity, reduced behavioral flexibility, impaired memory, disrupted sleep, and altered responses to alcohol (Crews et al., 2019). A study using MRI diffusion tensor imaging (DTI) to assess white matter structural integrity found AIE caused long-lasting changes in brain regional diffusivity and fractional anisotropy that correlated positively with diminished object recognition memory (Vetreno et al., 2013). A weakness of the current study is that we do not have imaging or behavioral data to relate to changes in protein expression in this group of AIE treated rats. Other models of adolescent alcohol exposure have reported damage to PFC-fmi myelin in rats (Vargas et al., 2014) as well as mice (Rice et al., 2019). Rice and colleagues found a more modest adolescent alcohol exposure in mice than ours studied here in rats, and reduced PFC MBP + IHC just after AIE exposure similar to our finding in PFC IL, although PFC subregions were not assessed (Rice et al., 2019). MRI imaging studies find AIE causes long-lasting changes in adult corpus callosum white matter volume (Ehlers et al., 2013b) as well as diffusivity (Vetreno et al., 2016). *In vivo* MRI finds AIE reduces cortical-cortical and cortico-limbic resting state functional connectivity (Broadwater et al., 2018) that could be related to white matter damage. Pascual et al. (2014) indicated that neuroimmune activation in the PFC contributed to neuroinflammation and demyelination processes following adolescent ethanol exposure. They reported that the important myelin fiber disruptions induced by chronic alcohol in adult mice were associated with the activation of TLR4 (Toll-like receptor 4) receptors and neuroinflammation in the cerebral cortex and cc (Alfonso-Loeches et al., 2012). Previous studies have found that AIE exposure induces multiple proinflammatory cytokines, TLRs and RAGE, as well as HMGB1, an agonist at these immune receptors in the PFC of the rat and human alcoholics (Vetreno and Crews, 2012; Vetreno et al., 2013), which persists after AIE exposure and increases during maturation to young adulthood (Vetreno and Crews, 2012). Although the association of AIE-induced neuroimmune gene expression is not clear, we report here AIE increased microglial Iba1+IHC in PrL and increased NG2+/Iba1+ in fmi and SVZ. AIE induces a loss of adult behavioral flexibility consistent with altered PFC function (Crews et al., 2016; Crews et al., 2019). Our findings of AIE-induced changes in adult PFC OPC and OL protein expression suggest white matter and myelin damage may contribute to AIE-induced deficits in PFC functions.

The most robust changes induced by AIE were in OPC markers. In fmi white matter, we found AIE caused large increases in NG2+IHC just after ethanol exposure at P57 that persisted without additional ethanol treatment to at least P95, consistent with a long-lasting persistent increase in NG2 glia in PFC white matter. NG2 glia proliferate in response to cytokines, growth factors, neuronal activation (Moyon et al., 2016), and following ethanol self-administration (He et al., 2009). Microglial marker Iba1 is increased in multiple brain regions in the post-mortem brain of individuals with alcohol use disorder (He and Crews, 2007). AIE increases brain Toll-like receptor expression and proinflammatory cytokines and induces a hyper-ramified morphology of microglia with increases in CD11b expression (Crews et al., 2017a; Crews et al., 2017b; Walter et al., 2017). Previous studies have found ethanol increases expression of NG2+IHC cells, including studies finding increased NG2 expression following ethanol is not lost in transgenic mice lacking TLR4 receptors (Alfonso-Loeches et al., 2012). Studies of NG2+IHC following striatal toxin-induced lesions find increases in proliferation over 3–4 weeks of recovery that are associated with the emergence of newly formed NG2+/Iba1+ positive cells (He and Crews, 2008; Jin et al., 2018). These studies did not distinguish striatal gray vs white matter. It is possible that microglia contribute to induction of PFC NG2 proliferation and/or NG2 and/or that NG2 and microglia may share progenitors. In PFC white matter fmi, we found AIE increased NG2+IHC, but not Iba1+IHC, while increasing NG2+/Iba1+ co-localization. In PFC PrL gray matter, NG2+IHC did not change whereas Iba1+IHC increased slightly, but co-localization remained 10–20%. We assessed NG2+IHC in both PrL and IL PFC gray matter and found changes quite different from those in PFC fmi. In SVZ, AIE increased NG2+IHC, Iba1+IHC and co-localization. Previous studies have found AIE alters SVZ progenitors and increases Iba1+IHC as well as expression of proinflammatory cytokines (Crews et al., 2017a; Crews et al., 2019). These findings suggest NG2+ progenitors are altered by AIE across PFC regions. Interestingly, in neurogenic SVZ, NG2+IHC was not changed just after AIE exposure. However, controls showed a marked maturational decline in NG2+IHC such that at P95, AIE exposure increased NG2+IHC and NG2+/Iba1+ positive cells but not Olig2+IHC cells, consistent with AIE-induced injury increasing NG2+ progenitor pool differentiation into microglial-like cells.

Previous studies in a rat model of alcohol dependence found alcohol suppressed SVZ stem cell proliferation, and abstinence led to a burst of proliferation followed by a persistent decline in doublecortin, a marker of neurogenesis (Hansson et al., 2010). Similarly, AIE, as studied here, causes a persistent decrease in adult SVZ proliferation and neurogenesis (Liu and Crews, 2017) that is opposite from the increases in NG2+IHC we report here, possibly due to a shift from neuronal differentiation to NG2+ glial differentiation. NG2+ glia may increase due to increased neuroimmune signaling, AIE-induced myelin damage, and/or alterations in SVZ neuroprogenitor differentiation. Our finding that AIE alters NG2+IHC in fmi and SVZ, but not PFC gray matter, is consistent with AIE white matter damage and increased proinflammatory gene expression leading to persistent changes in adult NG2+IHC cells and NG2+/Iba1+ cells. AIE has been found to cause long-lasting increases in proinflammatory gene expression. Further, humans with AUD generally start alcohol abuse early in life and have increased cortical Iba1+IHC and proinflammatory gene expression in PFC (Crews and Vetreno, 2016). Taken together, these studies are consistent with AIE increasing proinflammatory gene expression and damaging PFC, which increases NG2+IHC cells primarily in PFC fmi white matter and SVZ, possibly due to an NG2+/Iba1+ subpopulation, while not altering PFC NG2+IHC cells in gray matter.

In summary, NG2+, PDGFR1α+, and Olig1+IHC identify glial progenitor populations that continue to show modest developmental declines between late adolescence (i.e., P57) and mature adult rats (i.e., P95) in frontal white matter fmi, PFC gray matter, and neurogenic SVZ. AIE exposure increased NG2+IHC glia in white matter fmi and neurogenic SVZ, but not gray matter. AIE also increased PFC NG2+/Iba1+ cells in white matter fmi and neurogenic SVZ, whereas only Iba1+IHC cells were increased in gray matter by AIE, consistent with neuroimmune signaling impacting NG2+ glial OPCs and OLs in the PFC. OL marker proteins change with maturation and following AIE in PFC white matter fmi, gray matter, and progenitor-rich SVZ. The changes in OL proteins are consistent with OL remodeling by age as well as by AIE.

## Data Availability

The raw data supporting the conclusions of this article will be made available by the authors, without undue reservation.

## Abbreviations

AIE, adolescent intermittent ethanol; ANOVA, analysis of variance; AUD, alcohol use disorder; BEC, blood ethanol concentration; CNPase, CNP-2,3-cyclic nucleotide 3-phosphodiesterase; DAB, nickel-enhanced diaminobenzidine; DTI, diffusion tensor magnetic resonance imaging; fmi, forceps minor of the corpus callosum; i.g. intragastric; IL, infralimbic cortex; +IHC, positive immunohistochemistry; MAG, myelin-associated glycoprotein; MBP, myelin basic protein; MOG, myelin oligodendrocyte glycoprotein; mPFC, medial prefrontal cortex; MRI, magnetic resonance imaging; mRNA, messenger RNA; Olig, oligodendrocyte transcription factor; OLs, oligodendrocytes; OPCs, oligodendrocyte progenitor cells; P, postnatal day; PBS, phosphate-buffered saline; PCR, polymerase chain reaction; PDGF, platelet-derived growth factor; PDGFRs, PDGF receptors; PFC, prefrontal cortex; PLP, proteolipid protein; PrL, prelimbic cortex; RNA, ribonucleic acid; RT-PCR, reverse transcription-PCR; SVZ, subventricular zone; TLRs, Toll-like receptors.
