# Supplementary material for: Expression of Oligodendrocyte and Oligoprogenitor Cell Proteins in Frontal Cortical White and Gray Matter: Impact of Adolescent Development and Ethanol Exposure
Source: Front Pharmacol. 2021 May 6;12:651418. doi: 10.3389/fphar.2021.651418 (PMC8134748; doi:10.3389/fphar.2021.651418)
Supplement: Supplementary file 1 [file datasheet1.docx]

| **Antibodies** | **Isotype** | **Source/**  **Purification** | **Dilution** | **Source** | **Validation** |
| --- | --- | --- | --- | --- | --- |
| **Olig1** | **Rabbit IgG** | **Polyclonal** | **1:1200** | **Abcam Inc., Cambridge, MA, USA (ab106648)** | **IHC** |
| **Olig2** | **Rabbit IgG** | **Polyclonal** | **1:1600** | **Abcam Inc., Cambridge, MA, USA (ab136253)** | **IHC, WB, ICC, IP, CHIPseq** |
| **NG2** | **Rabbit IgG** | **Polyclonal** | **1:1000** | **EMD Millipore, Billerica, MA, USA (AB5320)** | **IHC, WB** |
| **NG2** | **Rabbit IgG** | **Polyclonal** | **1:1000** | **Abcam Inc., Cambridge, MA, USA (ab40390)** | **IHC, WB, ICC/IF** |
| **MBP** | **Rabbit IgG** | **Polyclonal** | **1:400** | **Abcam Inc., Cambridge, MA, USA (ab40390)** | **IHC, WB, ICC/IF** |
| **PLP** | **Rabbit IgG** | **Polyclonal** | **1:2000** | **Novus Biologicals, Littleton, CO, USA (NB100-74503)** | **IHC, WB, ICC/IF** |
| **MOG** | **Rabbit IgG** | **Monoclonal** | **1:100** | **Novus Biologicals, Littleton, CO, USA (NBP1-95505)** | **IHC, WB, ICC/IF** |
| **MAG** | **Mouse IgG1** | **Monoclonal** | **1:4000** | **Abcam Inc., Cambridge, MA, USA (ab89780)** | **IHC, WB, Flow Cyt, ELISA, ICC/IF** |
| **PDGF Rα** | **Goat IgG** | **Polyclonal** | **1:800** | **R&D systems, Inc., Minneapolis, MN, USA (AF1062)** | **IHC, WB** |
| **CNPase** | **Mouse IgG** | **Monoclonal** | **1:1000** | **Abcam Inc., Cambridge, MA, USA (ab6319)** | **IHC, WB, Flow Cyt, ELISA, ICC/IF** |
| **Iba1** | **Mouse IgG1** | **Monoclonal** | **1:1000** | **ThermoFisher Scientific, USA (MA5-27726)** | **IHC, WB, Flow Cyt, ICC/IF** |

| **Supplemental Table 2. List of primer sequences for RTPCR analysis.** | | |
| --- | --- | --- |
| Primer | Forward | Reverse |
| *NG2* | GAACCGAAAGGCTCGCTTTG | GTCGTTGACGGGGTTTACCT |
| *MBP* | GTGGGGGTAAGAGAAACGCA | CAAGGTCGGTCGTTCAGTCA |
| *MOG* | AGAGCTAAGAAACCCCTTTTGAG | CCTGATAGACTCCCTGGCCT |
| *CNPase* | AGACATAGTGCCCGCAAAG | GTCCAGACGCTTGTACTCCT |
| *β actin* | CTACAATGAGCTGCGTGTGGC | CAGGTCCAGACGCAGGATGGC |
|  |  |  |
